# Supplementary material for: Radical and lunatic fringes modulate notch ligands to support mammalian intestinal homeostasis
Source: eLife. 2018 Apr 9;7:e35710. doi: 10.7554/eLife.35710 (PMC5896954; doi:10.7554/eLife.35710)
Supplement: Supplementary file 1. — List of antibodies used for Immunofluorescence and Western blotting. [file elife-35710-supp1.docx]

| Primary Antibody | Supplier | Catalogue # | RRID # | Dilution* |
| --- | --- | --- | --- | --- |
| anti-β-ACTIN | Abcam | ab6276 | RRID:AB_2223210 | 1:4000 (WB) |
| Anti-CD24 (APC) | Abcam | ab51535 | RRID:AB_2072741 | 1:500 (FC) |
| Anti-DLL1 | Abcam | ab85346 | RRID:AB_1860332 | 1:500 (WB) |
| Anti-DLL4 | Abcam | ab7280 | RRID:AB_449562 | 1:1000 (WB) |
| Anti-GFP | Abcam | ab5450 | RRID:AB_304897 | 1:200 (IF) (IF-p) |
| Anti-HES1 | Abcam | ab108937 | RRID:AB_10862625 | 1:1000 (WB) |
| Anti-HES5 | Santa Cruz Biotechnology | sc-25395 | RRID:AB_2118099 | 1:500 (WB) |
| Anti-HEY1 | Abcam | ab154077 |  | 1:1000 (WB) |
| Anti-JAG1 | Santa Cruz Biotechnology | sc-6011 | RRID:AB_649689 | 1:500 (WB) |
| Anti-KRT20 | Origene | TA300936 | RRID:AB_2265515 | 1:1000 (FC) |
| Anti-LFNG | Santa Cruz Biotechnology | sc-324092 |  | 1:1000 (WB)  1:500 (ELISA) |
| Anti-LYSOZYME | Abcam | ab108508 | RRID:AB_10861277 | 1:100 (IF) |
| Anti-MFNG | Santa Cruz Biotechnology | sc-292668 | RRID:AB_11149012 | 1:1000 (WB)  1:500 (ELISA) |
| Anti-MUC2 | Abcam | ab76774 | RRID:AB_1523987 | 1:100 (IF) |
| Anti-MUC2 | Santa Cruz Biotechnology | sc-15334 | RRID:AB_2146667 | 1:200 (IF-p) |
| Anti-RFNG | Santa Cruz Biotechnology | sc-8240 | RRID:AB_2178618 | 1:1000 (WB)  1:500 (ELISA) |
| Anti-CK20 | Abcam | ab118574 | RRID:AB_10901782 | 1:200 (IF-p) |
| Anti-Ki-67 | Abcam | ab15580 | RRID:AB_443209 | 1:200 (IF-p) |
| Anti-CHGA | Abcam | ab45179 |  | 1:50 (IF-p) |
| Anti-DCAMKL1 | Abcam | ab37994 |  | 1:200 (IF-p) |
| Anti-NICD (Val1744) | Cell Signalling Technology | 4147 | RRID:AB_2153348 | 1:600 (IF-p) |

**SUPPLEMENTARY METHODS**

**Antibodies used:**

***Application: IF: (Immunofluorescence); WB: (Western Blotting); FC: (Flow Cytometry); IF-p: (IF on paraffin embedded sections)**
